# Serological Evidence for the Association Between Epstein-Barr Virus Infection and Sjögren’s Syndrome

**DOI:** 10.3389/fimmu.2020.590444

**Published:** 2020-10-30

**Authors:** Jingxiu Xuan, Zhiqian Ji, Bin Wang, Xiaoli Zeng, Rongjuan Chen, Yan He, Peishi Rao, Puqi Wu, Guixiu Shi

**Affiliations:** ^1^ Department of Rheumatology and Clinical Immunology, The First Affiliated Hospital of Xiamen University, Xiamen, China; ^2^ Department of Science & Technology, Xiamen Key Laboratory of Rheumatology and Clinical Immunology, Xiamen, China

**Keywords:** Epstein-Barr virus, Sjögren’s syndrome, systematic review, associations, meta-analysis

## Abstract

**Background:**

Exposure to Epstein-Barr virus (EBV) infection has been hypothesized to be an important risk factor for multiple rheumatic diseases, but the serological evidence so far for its role in Sjögren’s syndrome (SjS) is not clearly established yet. This study aimed to assess the seroepidemiological associations of antibodies to EBV with SjS.

**Methods:**

A seroepidemiological study containing 119 patients with SjS and 65 healthy controls was first performed, in which the associations of SjS with four commonly studied EBV antibodies including IgM-anti-viral capsid antigen (anti-VCA) antibody, IgG-anti-VCA antibody, IgG-anti-early antigen (anti-EA) antibody, and IgG-anti-EBV nuclear antigen 1 (anti-EBNA1) antibody were evaluated. A systematic review and meta-analysis of eligible seroepidemiological studies was also carried out, and data syntheses were performed using random-effect meta-analysis.

**Results:**

In the case-control study, the patients with SjS had both a significantly higher prevalence of IgG-anti-EA antibody positivity (31.9% vs. 3.1%, P < 0.001) and high titers of IgG-anti-EA antibody (P < 0.001) than healthy controls. The titer of IgG-anti-VCA antibody was significantly increased in the patients with SjS compared with healthy controls (P < 0.001). IgG-anti-EA antibody seropositive patients with SjS had lower levels of both C3 (P = 0.002) and C4 (P = 0.02), and the titer of IgG-anti-EA antibody was inversely related to the levels of both C3 (r = -0.31, P < 0.001) and C4 (r = -0.20, P = 0.03). A total of 14 eligible studies on the serological associations between EBV infection and SjS were finally included into the meta-analysis, which suggested obvious associations of SjS with IgM-anti-VCA antibody [Odds ratio (OR) = 5.77, 95%CI 1.73–19.25, P = 0.004] and IgG-anti-EA antibody (OR = 9.97, 95%CI 4.58-21.67, P < 0.00001).

**Conclusions:**

The findings from this study provide strong serological evidence for the association between EBV infection and SjS. SjS has obvious associations with IgM-anti-VCA antibody and IgG-anti-EA antibody. IgG-anti-EA antibody is linked to low levels of C3 and C4 in the patients with SjS, the significance of which needs to be addressed in further studies.

## Introduction

Sjögren’s syndrome (SjS) is a complex and heterogeneous rheumatic disease (1). SjS is characterized by autoantibody production and lymphocyte infiltration in exocrine glands such as salivary and lacrimal glands (2, 3). Exocrine glandular injuries induced by autoimmune attacks can gradually cause dryness of eyes and mouth (1). Like systemic lupus erythematosus (SLE) and rheumatoid arthritis (RA), the pathogenesis of SjS involves a complex interplay between genetic, immune and environmental factors, and the underlying molecular mechanisms remain to be defined (4–7). Though many studies have explored possible treatments of SjS, effective targeted therapies for SjS are still lacking (8, 9). Further studies are needed to elucidate SjS pathogenesis and find possible therapeutic targets.

Infections such as Epstein-Barr virus (EBV) infection have been proposed as one of the environmental triggers of SjS (10, 11). EBV is a common herpes virus affecting more than 90% of the total population worldwide (12). The possible roles of EBV infection in rheumatic diseases have been postulated for many years, and it has proven to be an important environmental trigger of several autoimmune diseases such as SLE and multiple sclerosis (MS) (13–16). Like SLE, a possible link between EBV infection and SjS has also been proposed for many years (17, 18). Increased prevalence of EBV infection and elevated viral loads in salivary glands had been reported in patients with SjS (19–21). There were also some studies exploring the serological evidence for the pathogenic role of EBV infection in SjS, and associations of SjS with EBV antibodies such as IgM-anti-viral capsid antigen (anti-VCA) antibody, IgG-anti-VCA antibody, IgG-anti-early antigen (anti-EA) antibody and IgG-anti-EBV nuclear antigen 1 (anti-EBNA1) antibody had been studied. However, the findings from these various studies were inconsistent (22–28). Therefore, the serological evidence for the contribution of EBV infection to the pathophysiology of SjS is not firmly established. To further explore the link between EBV antibodies and SjS, we performed an original seroepidemiological case-control study at first. We then carried out a systematic review and meta-analysis of available seroepidemiological studies on the associations between antibodies to EBV and SjS.

## Methods

### Participants

A seroepidemiological study using case-control design was performed to evaluate the association between EBV infection and SjS, in which the associations of SjS with EBV antibodies were analyzed. 119 patients with SjS were recruited in The First Affiliated Hospital of Xiamen University from June 2018 to December 2019. SjS was diagnosed according to the 2016 American College of Rheumatology (ACR) and the European League Against Rheumatism (EULAR) classification criteria for SjS (29). 65 healthy controls without autoimmune diseases were randomly recruited from individuals receiving routine medical examination in our hospital, whose age and gender matched SjS patients. The mean age of the SjS patients was 51.2 ± 15.7 years, and the mean age of the healthy controls was 50.7 ± 11.1 years (**Supplementary Table 1**). This study was approved by the Medical Ethics Committee at The First Affiliated Hospital of Xiamen University (KY202015-034). Written informed consent was obtained from all participants.

### Clinical Assessment

The disease activity of SjS patients was evaluated by the EULAR Sjögren’s syndrome disease activity index (ESSDAI) (30). Clinical data such as age, gender, disease history, clinical manifestations and treatment drugs were collected. Outcomes of laboratory tests such as anti-SSA/Ro, anti-SSB/La, C3, and C4 were also recorded.

### Detection of EBV Antibodies

Sera from SjS patients and healthy controls were stored at -80°C. Four commonly used antibodies to EBV including IgM-anti-VCA antibody, IgG-anti-VCA antibody, IgG-anti-EA antibody and IgG-anti-EBNA1 antibody were analyzed. EBV antibodies were detected by using commercial enzyme-linked immunosorbent assay (ELISA) kits according to the manufacturer’s instructions (EUROIMMUN, Germany). The cut-off values for positivity of IgG-anti-VCA antibody, IgG-anti-EA antibody and IgG-anti-EBNA1 antibody were defined as 22 RU/ml, and the cut-off value for IgM-anti-VCA antibody positivity was defined as a ratio of OD_Patient_/OD_Standard_ or OD_Control_/OD_Standard_ more than 1.1.

### Statistical Analysis

Quantitative variables were shown as mean ± standard deviation (SD) or median with interquartile range (IQR). Categorical variables were shown as counts with percentages. Differences in quantitative data between groups were compared by student’s t-test or Mann-Whitney U test when necessary. Differences in categorical variables between groups were compared by Chi-square or Fischer’s exact tests. Odds ratios (OR) with 95% confidence intervals (95%CI) were calculated to evaluate the associations between EBV antibodies and SjS. Data analyses were performed using GraphPad (Version 7.0, GraphPad Software, California, USA) and STATA (Version 12.0, StataCorp, Texas, USA). All tests were two-sided, and outcomes were considered statistically significant at P < 0.05.

### Systematic Review and Meta-Analysis

A systematic review and meta-analysis was performed to evaluate the relationship between antibodies to EBV and patients with SjS. We searched Pubmed and China National Knowledge Infrastructure (CNKI) from inception to May 16, 2020, to identify epidemiological studies on the serological associations between antibodies to EBV and SjS. We also searched the reference citations of those included studies to identify more possible articles. The following terms were used in the literature search: (Epstein-Barr virus OR EBV OR human herpesvirus 4 OR HHV-4) AND (Sjogren’s syndrome OR Sjögren’s syndrome OR Sjogren syndrome OR Sjögren syndrome). No language restriction was applied. Articles concerning the epidemiological associations of anti-EBV antibodies with SjS were reviewed. Studies eligible into the systematic review met the following criteria: 1) Clinical observational studies such as cohort studies, cross-sectional studies or case-control studies; 2) Participants contained at least 10 SjS patients; 3) Reporting data on the serological associations of EBV antibodies with SjS; 4) EBV antibodies were examined using recommended clinical laboratory methods such as immunofluorescence (IF) or ELISA; 5) Data did not overlap with other included studies. Studies not meeting the above eligible criteria were all excluded. Studies that did not specify the type of anti-EBV antibodies or did not provide usable data were excluded.

Two authors independently extracted data from eligible studies such as authors, country, sample size, types of EBV antibodies, laboratory methods, diagnostic criteria of SjS, cut-off values of seropositivity, and risk estimates with 95%CIs. Discrepancies in the data extracted by those two authors were resolved *via* group discussions among all authors.

Heterogeneity among included studies was assessed using the I^2^-statistic, and I^2^ more than 50% suggested high heterogeneity (31). To reduce the impact of heterogeneity on the pooled risk estimates, meta-analysis was performed by DerSimonian and Laird’s random-effect model (32). Publication bias was assessed by funnel plot. Review Manager (Version 5.2; Cochrane, London, United Kingdom) was used in statistical analyses, and P values less than 0.05 were considered statistically significant.

## Results

### Case-Control Study

The clinical characteristics of SjS patients and healthy controls in the case-control study are shown in **Supplementary Table 1**. There was no obvious difference in age and gender between SjS patients and healthy controls (P > 0.05). The mean ESSDAI of those 119 SjS patients was 2.1 ± 1.5.

**Figure 1:**
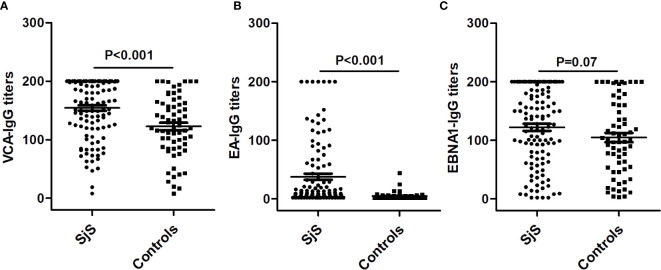
Differences in the titers of IgG-anti-VCA antibody, IgG-anti-EA antibody and IgG-anti-EBNA1 antibody between SjS patients and healthy controls. **(A)** The difference in the titers of IgG-anti-VCA antibody between SjS patients and healthy controls. **(B)** The difference in the titers of IgG-anti-EA antibody between SjS patients and healthy controls. **(C)** The difference in the titers of IgG-anti-EBNA1 antibody between SjS patients and healthy controls.

Compared with healthy subjects, SjS patients had both a significantly higher prevalence of IgG-anti-EA antibody positivity (31.9% vs. 3.1%, P < 0.001) and higher titers of IgG-anti-EA antibody (P < 0.001; **Table 1**). There was no obvious difference in the prevalence of IgM-anti-VCA antibody, IgG-anti-VCA antibody and IgG-anti-EBNA1 antibody between SjS patients and controls (P > 0.05; **Table 1**). However, the titer of IgG-anti-VCA antibody was significantly increased in SjS patients compared to healthy controls (P < 0.001; **Table 1**, **Figures 1A–C**). The titer of anti-EBNA1 IgG antibody was marginally increased in SjS patients than healthy controls (P = 0.07; **Table 1**).

**Table 1:** Associations between EBV antibodies and SjS in the case-control study.

EBV antibodies	SjS (n=119)	Controls (n=65)	P value
IgM-anti-VCA antibody			
Positivity (n, %)	3(2.5%)	0(0.0%)	0.55
IgG-anti-VCA antibody			
Antibody titers	166(123–200)	121(88–162)	<0.001*
Positivity (n, %)	117(98.3%)	62(95.4%)	0.35
IgG-anti-EA antibody			
Antibody titers	9(4–56)	3(2–4)	<0.001*
Positivity (n, %)	38(31.9%)	2(3.1%)	<0.001*
IgG-anti-EBNA1 antibody			
Antibody titers	128(69–186)	100(54–160)	0.07
Positivity (n, %)	107(89.9%)	56(86.2%)	0.44

*Bold values suggested statistically significant findings.

The clinical characteristics of SjS patients stratified by seropositivity status of EBV antibodies were then analyzed. As shown in the supplementary tables (**Supplementary Tables 2–5**), IgG-anti-EA antibody seropositive patients with SjS had lower levels of both C3 (P = 0.002) and C4 (P = 0.02). Among those 119 SjS patients, the titer of IgG-anti-EA antibody was inversely related to the levels of C3 (r = -0.31, P < 0.001) and C4 (r = -0.20, P = 0.03), and a marginally significant inverse correlation between IgG-anti-VCA antibody titer and C3 level was also observed (r = -0.17, P = 0.06) (**Table 2**). The titers of other EBV antibodies were not significantly related to the levels of C3 and C4 except for the marginally significant correlation between IgG-anti-VCA antibody and C3 level (P = 0.06; **Table 2**). The disease activity of SjS patients evaluated by ESSDAI was not significantly correlated with EBV antibodies (P > 0.05; **Table 2**).

**Table 2:** Correlations of EBV antibodies with clinical characteristics of SjS patients.

EBV antibodies	Spearman r	P value
IgG-anti-VCA antibody		
Age	0.16	0.08
ESSDAI	0.08	0.39
C3	-0.17	0.06
C4	-0.08	0.39
IgG-anti-EA antibody		
Age	0.08	0.37
ESSDAI	0.09	0.31
C3	-0.31	<0.001*
C4	-0.20	0.03*
IgG-anti-EBNA1 antibody		
Age	-0.05	0.58
ESSDAI	0.07	0.48
C3	-0.07	0.46
C4	-0.10	0.29

*Bold values suggested statistically significant findings.

### Systematic Review and Meta-Analysis

A total of 261 publications were found in the literature search. 224 studies were excluded after the initial evaluation by reading titles and abstracts. The rest of 37 studies were evaluated in details by reading full-texts, and 24 studies were further excluded as they did not analyze the epidemiological associations of EBV antibodies with SjS. Finally, 13 published studies focusing on the serological association between EBV infection and SjS were identified (22–28, 33–38). Among those 13 published studies, nine studies reported outcomes on IgG-anti-VCA antibody, seven studies reported outcomes on IgM-anti-VCA antibody, 6 studies reported outcomes on IgG-anti-EA antibody, and nine studies reported outcomes on IgG-anti-EBNA1 antibody (**Table 3**). Together with the present case-control study, a total of 14 available studies on the serological association between EBV infection and SjS were finally included into the meta-analysis (**Table 3**).

**Table 3:** Summarization of 14 studies focusing on the serological association between EBV infection and SjS.

Study	Country	Participants	Detection method	EBV antibodies
Venables PJ 1985 (22)	UK	26 SjS patients and 26 healthy controls	Indirect IF	IgG-anti-VCA antibody
Yamaoka K 1988 (33)	Japan	26 SjS patients and 15 healthy controls	Indirect IF	IgG-anti-VCA and IgM-anti-VCA antibodies
Venables PJ 1989 (25)	UK	20 SjS patients and 35 healthy controls	ELISA	IgG-anti-EBNA1 antibody
Inoue N 1991 (23)	Japan	32 SjS patients and 38 healthy controls	ELISA	IgG-anti-EBNA1 antibody
Yang J 1991 (34)	China	95 SjS patients and 8 healthy controls	Indirect IF	IgG-anti-VCA, IgM-anti-VCA and IgG-anti-EBNA1 antibodies
Marchini B 1994 (24)	Italy	12 SjS patients and 20 healthy controls	ELISA	IgG-anti-EBNA1 antibody
Toda I 1994 (26)	Japan	62 SjS patients and 47 healthy controls	Indirect IF	IgG-anti-VCA, IgG-anti-EA, and IgG-anti-EBNA1 antibodies
Gao C 2010 (35)	China	29 SjS patients and 44 healthy controls	ELISA	IgM-anti-VCA, IgG-anti-VCA and IgG-anti-EA antibodies
Li H 2014 (36)	China	75 SjS patients and 74 healthy controls	ELISA	IgM-anti-VCA, IgG-anti-VCA and IgG-anti-EA antibodies
Yang L 2015 (37)	China	39 SjS patients and 22 healthy controls	ELISA	IgG-anti-EBNA1 antibody
Zhang L 2019 (38)	China	72 SjS patients and 60 healthy controls	ELISA	IgM-anti-VCA, IgG-anti-VCA, IgG-anti-EA and IgG-anti-EBNA1 antibodies
Pasoto SG 2013 (27)	Brazil	100 SjS patients and 89 healthy controls	ELISA	IgM-anti-VCA, IgG-anti-VCA, IgG-anti-EA and IgG-anti-EBNA1 antibodies
Kivity S 2014 (28)	Israel	82 SjS patients and 139 healthy controls	Multiplexed assay	IgM-anti-VCA, IgG-anti-VCA, IgG-anti-EA and IgG-anti-EBNA1 antibodies
Present study	China	119 SjS patients and 65 healthy controls	ELISA	IgM-anti-VCA, IgG-anti-VCA, IgG-anti-EA and IgG-anti-EBNA1 antibodies

IF, immunofluorescence; ELISA, enzyme linked immunosorbent assay; SjS, Sjögren’s syndrome; VCA, viral capsid antigen; EA, early antigen; EBNA1, EBV nuclear antigen 1.

**Figure 2:**
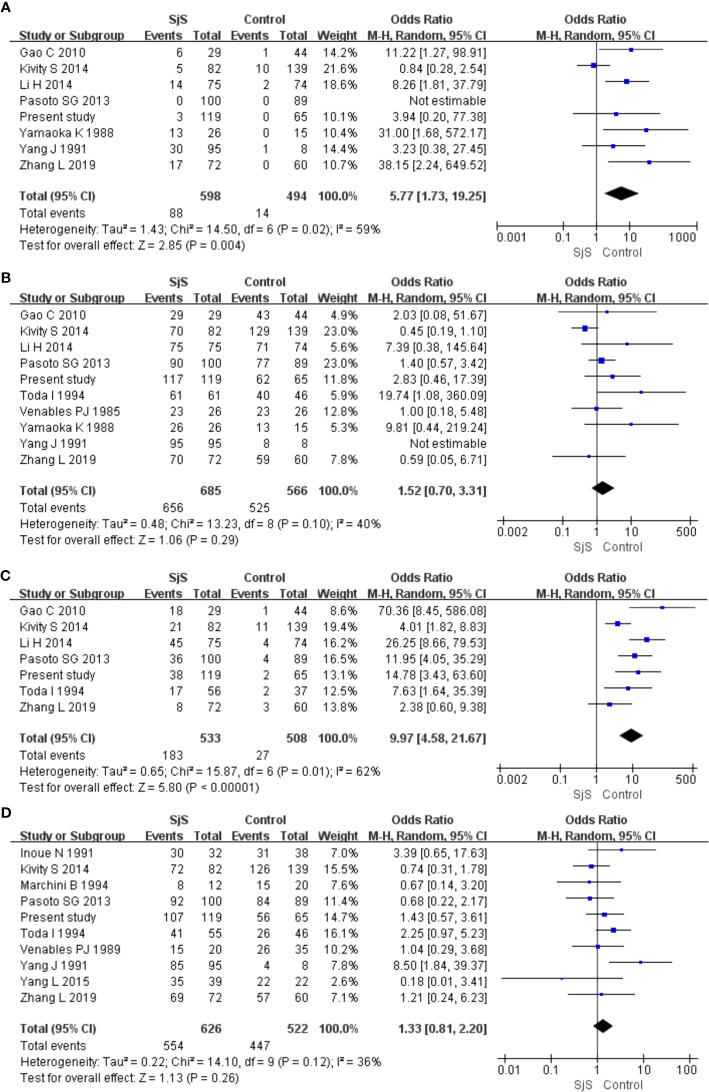
Forest plots in the meta-analysis of the associations between EBV antibodies and SjS. **(A)** Forest plot in the meta-analysis of the association between IgM-anti-VCA antibody and SjS. **(B)** Forest plot in the meta-analysis of the association between IgG-anti-VCA antibody and SjS. **(C)** Forest plot in the meta-analysis of the association between IgG-anti-EA antibody and SjS. **(D)** Forest plot in the meta-analysis of the association between IgG-anti-EBNA1 antibody and SjS.

Obvious heterogeneity was found among those eight studies on the association between IgM-anti-VCA antibody and SjS (I^2^ = 59%; **Figures 2A–D**). Meta-analysis of those eight studies suggested that IgM-anti-VCA antibody was significantly associated with SjS (Pooled OR = 5.77, 95%CI 1.73-19.25, P = 0.004) (**Figure 2A**). Obvious heterogeneity was also found among those seven studies on the association between IgG-anti-EA antibody and SjS (I^2^ = 62%), and meta-analysis of those seven studies revealed an obvious association between IgG-anti-EA antibody and SjS (Pooled OR = 9.97, 95%CI 4.58-21.67, P < 0.00001) (**Figure 2C**). However, obvious associations of SjS with IgG-anti-EBNA1 antibody and IgG-anti-VCA antibody were not found (P > 0.05; **Figures 2B, D**).

**Figure 3:**
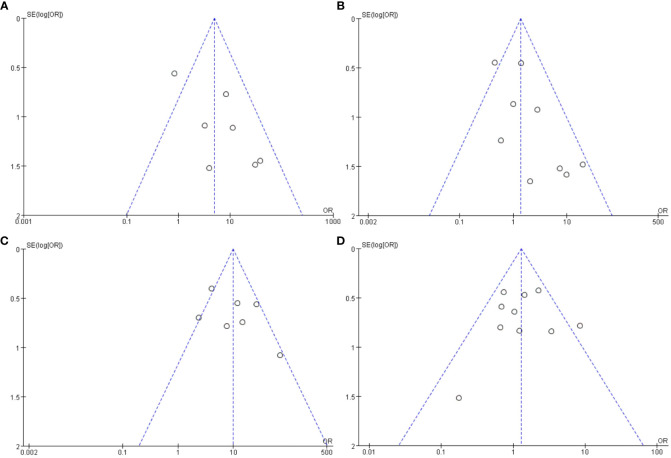
Funnel plots in the meta-analysis of the associations between EBV antibodies and SjS. **(A)** Funnel plot in the meta-analysis on IgM-anti-VCA antibody. **(B)** Funnel plot in the meta-analysis on IgG-anti-VCA antibody. **(C)** Funnel plot in the meta-analysis on IgG-anti-EA antibody. **(D)** Funnel plot in the meta-analysis on IgG-anti-EBNA1 antibody.

Funnel plots in this meta-analysis suggested low risk of publication bias, and all those 4 funnel plots were nearly symmetric (**Figure 3**).

## Discussion

Though EBV infection has been hypothesized to be a possible risk factor of SjS in a long term, prior studies have revealed conflicting results and the serological evidence is not yet definitely established (**Table 3**). For instance, half of those published studies on the association between IgM-anti-VCA antibody and SjS did not detect an obvious correlation, which may result from the low statistical power caused by the limited sample size. In the present study, we assessed the serological association between antibodies to EBV and SjS through both an original case-control study and a meta-analysis. The meta-analysis included a total of 14 studies, and could provide a more robust evaluation of the associations between antibodies to EBV and SjS by increasing sample size and statistical power. An obvious association of SjS with IgG-anti-EA antibody was observed in both our case-control study and the meta-analysis, and an obvious association of SjS with IgM-anti-VCA antibody was confirmed in the meta-analysis. However, IgG-anti-VCA antibody and IgG-anti-EBNA1 antibody were not associated with SjS in either the case-control study or the meta-analysis. Therefore, the above findings provide serological evidence for the association between EBV infection and SjS, and also scrutinize the different associations of SjS with different antibodies to EBV.

Multiple factors such as genetic factors, immunological abnormality and environmental risk factors are involved in the development of rheumatic diseases, and EBV infection has been proposed as an important environmental risk factor (39, 40). A meta-analysis by Hanlon et al. found that patients with SLE had significantly higher rates of IgG-anti-VCA antibody, IgA-anti-VCA antibody and IgG-anti-EA antibody than controls, but not IgG-anti-EBNA1 antibody (41). A recent study by Jog et al. revealed that EBV serological reactivation was associated with the probability of transitioning to SLE in at-risk individuals (42). A meta-analysis by Kudaeva et al. reported that the patients with RA had obviously higher rates of IgG-anti-VCA and IgG-anti-EA antibodies than controls, but not for IgG-anti-EBNA1 antibody (43). Therefore, there is compelling serological evidence for EBV infection as an important risk factor of SLE and RA. Our study revealed the obvious associations of SjS with IgM-anti-VCA antibody and IgG-anti-EA antibody *via* a systematic review and meta-analysis of 14 available studies, which added the evidence of EBV infection as an important environmental risk factor for SjS development.

There are some possible explanations for the discrepancy in the associations of SjS with different EBV antibodies. Molecular mimicry has been suggested to be a key mechanism underlying the role of EBV infection in triggering autoimmune diseases (44, 45). The sequence similarities or homologous peptides between human proteins and EBV viral proteins are different across those EBV viral antigens, and immune responses to different EBV viral antigens are distinct (46, 47). Different EBV viral antigens may have distinct cross reactivity with different autoantigens, and thus trigger the immune system to produce specific antibodies with distinct pathogenicity in SjS etiology, which may lead to the discrepancy in the associations of SjS with different EBV antibodies (48, 49). In addition, the non-significant associations of SjS with other EBV antibodies may result from the low statistical power caused by the relatively small sample size. For instance, though our study and one prior study indicated that the titer of IgG-anti-VCA antibody was significantly increased in patients with SjS compared with healthy controls (50), IgG-anti-VCA antibody positivity was not associated with SjS in both the original case-control study and the meta-analysis. Both patients with SjS and healthy controls have been found to have a high prevalence of IgG-anti-VCA antibody positivity (over 90%), and the sample size required to detect a significant association between IgG-anti-VCA antibody positivity and SjS in an epidemiological study is undoubtedly increased. To provide a more accurate estimate of the associations of SjS with EBV antibodies, further epidemiological studies on a large-scale are recommended.

An intriguing finding in our study is the inverse association between the titer of IgG-anti-EA antibody and the levels of C3 (r = -0.31, P < 0.001) and C4 (r = -0.20, P = 0.02) (**Table 2**). Low C3 or C4 levels can reflect disease severity of the patients with SjS, and are related to disease progression and poor outcomes (51–53). The lower levels of C3 and C4 in IgG-anti-EA antibody seropositive patients with SjS suggest that IgG-anti-EA antibody may have the possibility of promoting the progression of SjS by exhausting C3 and C4. Wilson et al. found that the serum cryoprecipitates from SjS patients contained autoantibodies to La and rheumatoid factor (RF) as well as complement proteins C3 and C4, which could activate classical complement pathway or alternative complement pathway (54). A key possible mechanism for the decreased C3 and C4 is the activation of complement system during EBV infection. In absence of antibodies, complement system may be activated with viral surface molecules and polysaccharides *via* alternative and mannose-lectin pathways during innate immunity (55, 56). In the presence of antibodies, antigen-antibody complexes formed by EBV antigens bound by antibodies can further activate complement system (57–59). Both C3 and C4 are the key components of the complement system, and can be exhausted by EBV-induced activation of the complement system. Furthermore, higher levels of IgG-anti-VCA antibody and IgG-anti-EA antibody have been proposed as indicators of chronic or frequent EBV reactivation, and EBV reactivation is intensively involved in the progression of EBV-related diseases (60–63). Frequent EBV reactivation can promote the production of EBV-related antibodies, which can further exhaust circulating complement proteins and result in decreased C3 and C4.

EBV can infect a variety of cell types such as B cells, epithelial cells, T cells and dendritic cells (64, 65). Published studies have revealed that high EBV viral loads and EBV-related antibodies exist in salivary glandular epithelial cells (SGECs) and saliva of SjS patients, and EBV antigens such as the lytic cycle antigen EA/D also exist in the SGECs of SjS patients (19, 66–68). Additionally, MHC-II molecules and viral early antigen have been found to be inappropriately expressed in SGECs of SjS patients, which may lead to vicious immune responses and promote the chronic inflammation in SGECs during SjS development (69). These studies imply that EBV can infect SGECs and trigger immune damages to salivary epithelium, which may promote the autoimmune processes in SjS pathogenesis.

EBV infection may be involved in SjS pathogenesis by promoting the activation of autoreactive B and T cells. EBV can infect B cells *via* envelope gp350/220 binding to the complement receptor type 2 (CR2) on B cells and *via* gp42 interacting with HLA class II molecules on B cells (64, 70, 71). The binding of the complex of C3d and EBV or antigen-antibody complexes to B cells brings CD19 into proximity of BCR-associated kinases, and the cytoplasmic tail of CD19 rapidly becomes tyrosine-phosphorylated, which can further promote the proliferation and activation of B cells (72–75). Some EBV proteins such as LMP2A and LMP1 have proven to prevent infected B cells from apoptosis and thus promote the progression of autoimmunity (76–79). Some studies have suggested that EBV can alter the differentiation and interrupt the normal function of T cells through different mechanisms, which may contribute to autoimmunity development (80–83). Therefore, EBV infection can cause the abnormal activation of B cells and/or T cells, which can further result in loss of immune tolerance and the development of autoimmunity (84–87). In SjS, active EBV infection has been found to be related to ectopic lymphoid structures (ELS) in the glandular tissues of SjS patients, suggesting that it may drive local autoimmune response and the activation of autoreactive B cells during the progression of SjS (88, 89).

There are other explanations for the roles of EBV infection in the pathogenesis of SjS (11). One explanation is molecular mimicry between EBV proteins and self antigens, which may promote the development of SjS through inducing the formation of cross-reactive autoantibodies to both pathogens and self-antigens (90–93). For instance, a study by Navone et al. found that antibodies to EBV could recognize autoantigens such as alpha-fodrin and tear lipocalin (94). Moreover, EBV can directly regulate innate immune cells such as dendritic cells (95, 96). An activated interferon-α (IFN-α) signature pathway is involved in the autoimmune process of SjS, and EBV DNA and RNA have been reported to activate plasmacytoid dendritic cells (pDC) through engagement of Toll-like receptor 9 (TLR-9) and TLR-7, and then increase IFN-α production (95, 96). To date, there is no confirming evidence to verify the pathogenic role of EBV in SjS pathogenesis, and the link remains to be further elaborated.

Several limitations exist in the present study. A major limitation is the retrospective case-control design in those included studies, which was unable to adequately evaluate the causality between antibodies to EBV and SjS. Further studies using a prospective design are recommended. Besides, the sample size of some included studies was limited, and owing to the limited sample size of recruited participants, the influence of other confounding factors such as treatment drugs on the associations of anti-EBV antibodies with SjS was not excluded. Finally, the cut-off values determining seropositivity of anti-EBV antibodies were different among the included studies, which may result in obvious heterogeneity in the meta-analysis and impair the strength of the findings.

In summary, the findings from this study provide strong serological evidence for the association between EBV infection and SjS. Moreover, anti-EA-IgG antibody is likely linked to low levels of C3 and C4 in patients with SjS. More efforts in addressing the roles of EBV infection in SjS and the underlying molecular mechanisms are needed.

## Data Availability Statement

The raw data supporting the conclusions of this article will be made available by the authors, without undue reservation.

## Ethics Statement

The studies involving human participants were reviewed and approved by Medical Ethics Committee at The First Affiliated Hospital of Xiamen University. The patients/participants provided their written informed consent to participate in this study.

## Author Contributions

GS and JX designed the study and wrote the manuscript. ZJ and BW analyzed data and wrote the manuscript. XZ, RC, PR, YH, and PW collected samples and data. All authors contributed to the article and approved the submitted version.

## Funding

This work was supported by grants from the National Natural Science Foundation of China (Grant No. 81971536 and No. U1605223) to GS, the First Affiliated Hospital of Xiamen University Projects for Young Scholar, China Funding (No. XYY2016013) to ZJ, and the National Natural Science Foundation of China (No.81701556) to YH.

## Conflict of Interest

The authors declare that the research was conducted in the absence of any commercial or financial relationships that could be construed as a potential conflict of interest.

## Abbreviations

anti-EA, anti-early antigen; anti-EBNA1, anti-EBV nuclear antigen 1; anti-VCA, anti-viral capsid antigen; CR2, complement receptor type 2; CNKI, China National Knowledge Infrastructure; EBV, Epstein-Barr virus; ELISA, enzyme-linked immunosorbent assay; ELS, ectopic lymphoid structures; ESSDAI, European League Against Rheumatism Sjögren’s syndrome disease activity index; IF, immunofluorescence; IFN-α, interferon-α; IQR, interquartile range; MS, multiple sclerosis; OR, Odds ratios; pDC, plasmacytoid dendritic cells; RA, rheumatoid arthritis; RF, rheumatoid factor; SD, standard deviation; SGECs, salivary glandular epithelial cells; SjS, Sjögren’s syndrome; SLE, systemic lupus erythematosus; TLR-9, Toll-like receptor 9; 95%CI, 95% confidence interval.
