# Supplementary material for: Serological Evidence for the Association Between Epstein-Barr Virus Infection and Sjögren’s Syndrome
Source: Front Immunol. 2020 Oct 30;11:590444. doi: 10.3389/fimmu.2020.590444 (PMC7662096; doi:10.3389/fimmu.2020.590444)
Supplement: Supplementary file 1 [file DataSheet_1.doc]

Supplementary table 1 Characteristics of SjS patients and healthy controls in the case-control study

| **Items** | **SjS (n = 119)** | **Controls (n = 65)** | **P values** |
| --- | --- | --- | --- |
| Gender (Female, %) | 110 (92.4%) | 60 (92.3%) | 0.98 |
| Age (Years, Mean ± SD) | 51.2 ± 15.7 | 50.7 ± 11.1 | 0.81 |
| ESSDAI (Mean ± SD) | 2.1 ± 1.5 | --^#^ | -- |
| Anti-SSA positivity (n, %) | 94 (79.0%) | -- | -- |
| Anti-SSB positivity (n, %) | 45 (37.8%) | -- | -- |
| With low C3 (n, %) | 49 (41.2%) | -- | -- |
| With low C4 (n, %) | 17 (14.3%) | -- | -- |

(^#^data were not available.)

**Supplementary Table 2 Characteristics of SjS patients stratified by IgM-anti-VCA seropositivity status**

| **Items** | **Positive (n = 3)** | **Negative (n=116)** | **P values** |
| --- | --- | --- | --- |
| Gender (Female, %) | 3 (100.0%) | 107 (92.2%) | 1.00 |
| Age (Years, Mean ± SD) | 56.7 ± 10.0 | 51.0 ± 15.8 | 0.54 |
| ESSDAI (Mean ± SD) | 2.7 ± 1.2 | 2.1 ± 1.5 | 0.51 |
| Anti-SSA positivity (n, %) | 2 (66.7%) | 92 (79.3%) | 0.51 |
| Anti-SSB positivity (n, %) | 1 (33.3%) | 44 (37.9%) | 1.00 |
| With low C3 (n, %) | 2 (66.7%) | 47(40.5%) | 0.57 |
| With low C4 (n, %) | 2 (66.7%) | 15 (12.9%) | 0.05 |
| C3 level (g/L) | 0.89 ± 0.19 | 0.94 ± 0.28 | 0.73 |
| C4 level (g/L) | 0.09 ± 0.06 | 0.20 ± 0.10 | 0.09 |

**Supplementary table 3 Characteristics of SjS patients stratified by IgG-anti-VCA seropositivity status**

| **Items** | **Positive (n = 117)** | **Negative (n = 2)** | **P values** |
| --- | --- | --- | --- |
| Gender (Female, %) | 108 (92.3%) | 2 (100.0%) | 1.00 |
| Age (Years, Mean ± SD) | 51.7 ±15.2 | 23.5 ± 23.3 | 0.01 |
| ESSDAI (Mean ± SD) | 2.1 ± 1.5 | 0.5 ± 0.7 | 0.13 |
| Anti-SSA positivity (n, %) | 92 (78.6%) | 2 (100.0%) | 1.00 |
| Anti-SSB positivity (n, %) | 43 (36.8%) | 2 (100.0%) | 0.14 |
| With low C3 (n, %) | 49 (41.9%) | 0 (0.0%) | 0.51 |
| With low C4 (n, %) | 17 (14.5%) | 0 (0.0%) | 1.00 |
| C3 level (g/L) | 1.15 ± 0.03 | 0.94 ± 0.28 | 0.29 |
| C4 level (g/L) | 0.19 ± 0.03 | 0.19 ± 0.10 | 0.97 |

**Supplementary table 4 Characteristics of SjS patients stratified by IgG-anti-EA seropositivity status**

| **Items** | **Positive (n = 38)** | **Negative (n = 81)** | **P values** |
| --- | --- | --- | --- |
| Gender (Female, %) | 35 (92.1%) | 75 (92.6%) | 0.93 |
| Age (Years, Mean ± SD) | 53.5 ± 12.5 | 50.1 ± 16.9 | 0.27 |
| ESSDAI (Mean ± SD) | 2.5 ± 1.6 | 1.9 ± 1.5 | 0.07 |
| Anti-SSA positivity (n, %) | 30 (78.9%) | 64 (79.0%) | 0.99 |
| Anti-SSB positivity (n, %) | 14 (36.8%) | 31 (38.3%) | 0.88 |
| With low C3 (n, %) | 22 (57.9%) | 27 (33.3%) | **0.01** |
| With low C4 (n, %) | 10 (26.3%) | 7 (8.6%) | **0.01** |
| C3 level (g/L) | 0.83 ± 0.34 | 1.00 ± 0.23 | **0.002** |
| C4 level (g/L) | 0.16 ± 0.09 | 0.21 ± 0.11 | **0.02** |

**Supplementary table 5 Characteristics of SjS patients stratified by IgG-anti-EBNA1 seropositivity status**

| **Items** | **Positive (n= 107)** | **Negative (n = 12)** | **P values** |
| --- | --- | --- | --- |
| Gender (Female, %) | 9 (8.4%) | 0 (0.0%) | 0.60 |
| Age (Years, Mean ± SD) | 50.9 ± 15.5 | 53.5 ± 17.9 | 0.59 |
| ESSDAI (Mean ± SD) | 2.1 ± 1.5 | 1.8 ± 1.2 | 0.40 |
| Anti-SSA positivity (n, %) | 86 (80.4%) | 8 (66.7%) | 0.27 |
| Anti-SSB positivity (n, %) | 39 (36.4%) | 6 (50.0%) | 0.36 |
| With low C3 (n, %) | 44 (41.1%) | 5 (41.7%) | 0.97 |
| With low C4 (n, %) | 16 (15.0%) | 1 (8.3%) | 1.00 |
| C3 level (g/L) | 0.94 ± 0.28 | 0.97 ± 0.20 | 0.69 |
| C4 level (g/L) | 0.19 ± 0.11 | 0.21 ± 0.07 | 0.68 |
